# Comparison of short-term and medium-term outcomes between intracorporeal anastomosis and extracorporeal anastomosis for laparoscopic left hemicolectomy

**DOI:** 10.1186/s12957-022-02735-7

**Published:** 2022-08-27

**Authors:** Li-Ming Wang, Bor-Kang Jong, Chun-Kai Liao, Ya-Ting Kou, Yih-Jong Chern, Yu-Jen Hsu, Pao-Shiu Hsieh, Wen-Sy Tsai, Jeng-Fu You

**Affiliations:** Division of Colon and Rectal Surgery, Department of Surgery, Chang Gung Memorial Hospital at Linkou, Chang Gung University College of Medicine, No. 5, Fuxing Street, Guishan District, Taoyuan City, Taiwan

**Keywords:** Intracorporeal anastomosis, Extracorporeal anastomosis, Laparoscopy surgery, Left hemicolectomy, Disease-free survival

## Abstract

**Background:**

Few studies have evaluated the feasibility and safety of intracorporeal anastomosis (IA) for left hemicolectomy. Here, we aimed to investigate the potential advantages and disadvantages of laparoscopic left hemicolectomy with IA and compare the short- and medium-term outcomes between IA and extracorporeal anastomosis (EA).

**Methods:**

We retrospectively analyzed 133 consecutive patients who underwent laparoscopic left hemicolectomies from July 2016 to September 2019 and categorized them into the IA and EA groups. Patients with stage 4 disease and conversion to laparotomy or those lost to follow-up were excluded. Postoperative outcomes between IA and EA groups were compared. Short-term outcomes included postoperative pain score, bowel function recovery, complications, duration of hospital stay, and pathological outcome. Medium outcomes included overall survival and disease-free survival for at least 2 years.

**Results:**

After excluding ineligible patients, the remaining 117 underwent IA (*n* = 40) and EA (*n* = 77). The IA group had a shorter hospital stay, a shorter time to tolerate liquid or soft diets, and higher serum C-reactive protein level on postoperative day 3. There was no difference between two groups in operative time, postoperative pain, specimen length, or nearest margin. A 2-year overall survival (IA vs. EA: 95.0% vs. 93.5%, *p* = 0.747) and disease-free survival (IA vs. EA: 97.5% vs. 90.9%, *p* = 0.182) rates were comparable between two groups.

**Conclusions:**

Laparoscopic left hemicolectomy with IA was technically feasible, with better short-term outcomes, including shorter hospital stays and shorter time to tolerate liquid or soft diets. The IA group had higher postoperative serum C-reactive protein level; however, no complications were observed. Regarding medium-term outcomes, the overall survival and disease-free survival rates were comparable between IA and EA procedures.

## Background

Laparoscopic techniques have been in clinical practice since the 1980s. These approaches were initially used by general surgeons for laparoscopic cholecystectomy and subsequently adapted for colorectal surgery in 1991 [1]. Compared to conventional open surgery methods, laparoscopic colorectal surgery has better short-term outcomes, including reductions in perioperative mortality, wound complications, blood loss, and postoperative pain. Additionally, faster resumption of oral diets and shorter hospital stays have also been reported [2–5]. Although the short-term outcomes of minimally invasive colorectal surgery are more favorable than those of open surgery, both approaches have comparable long-term outcomes [6, 7].

With the development of minimally invasive surgical techniques, laparoscopic colorectal resection is currently the mainstream approach for colorectal cancer surgery. After more than two decades of laparoscopic surgery, various methods of intestinal anastomosis other than laparoscopic colorectal resection have gradually been devised. Initially, intestinal anastomosis was often performed with extracorporeal anastomosis (EA). However, as this technique matured, intracorporeal anastomosis (IA) was adopted. The IA strategy involves opening the bowel intracorporeally during surgery, potentially dislodging bowel contents and tumor cells, which might lead to undesirable consequences.

Several studies have demonstrated the advantages of IA over EA for laparoscopic right hemicolectomy. Total laparoscopic right colectomy with IA was associated with shorter postoperative hospital stays, comparable short-term outcomes, and potential acquisition of more lymph nodes for better accuracy of oncological evaluations [8–10].

However, few studies have reported the feasibility and safety of IA for left hemicolectomy. Here, we aimed to investigate the potential advantages and disadvantages of laparoscopic left hemicolectomy using IA. The primary objective of this study was to examine the short-term outcomes, including postoperative pain score, bowel function recovery, complications, hospital stay duration, and pathological outcome, between IA and EA in laparoscopic left hemicolectomy. The secondary objective was to compare the medium-term outcomes, including overall survival and disease-free survival for at least 2 years between these two approaches.

## Methods

### Patient selection

From July 2016 to September 2019, 133 patients with colon cancer who underwent laparoscopic left hemicolectomy at the Linkou Chang Gung Memorial Hospital were retrospectively analyzed. All patients had confirmed cancer diagnosis via colonoscopic biopsy before operation. Twelve patients with stage 4 disease and two patients who were converted to laparotomy were excluded.

### Data collection

Detailed patient data were retrieved from the Colorectal Section Tumor Registry of the Chang-Gung Memorial Hospital. A prospectively designed database of postoperative records of patients, who were consecutively and actively followed up, was generated. This study was approved by the Institutional Review Board of our hospital (IRB no. 202000644B0).

Patient data, including age, sex, body mass index (BMI), and preoperative albumin level at 1 week before surgery, were analyzed. Additionally, operative details, including operation time, operation method, anastomosis method (IA vs. EA), and blood loss, were recorded. Pathological parameters, including tumor location, tumor size, number of harvested lymph nodes, specimen length, and nearest length from the tumor to the margin, T stage, and N stage, were examined. Outcomes after surgery were also analyzed. Short-term outcomes included pain scores after the surgery from the operation day to postoperative day 3 based on a numeric rating scale (NRS) in nursing records and recovery parameters, such as time to first flatus, time to first stool passage, and time to tolerate liquid and soft diets. Complications, mortality, hospital stay duration, and readmission within 30 days of discharge were also analyzed.

All patients were followed up for at least 2 years, and the last follow-up date in this study was March 5, 2022. Physicians in our department adopted similar follow-up routines and adjuvant treatment protocols. All patients were evaluated weekly by a multidisciplinary team to determine the actual cancer stage according to clinical information and pathology reports. However, the final decisions regarding adjuvant chemotherapy administration were made according to each physician’s opinion and each patient’s choice. After primary resection, all patients participated in a follow-up program that included outpatient visits every 3–6 months for physical examinations and evaluations of carcinoembryonic antigen (CEA) level. Additionally, chest radiography, abdominal sonography, abdominal computed tomography scan, and colonoscopy were performed postoperatively every 1–2 years. The first recurrence date was defined as the first date when the existence of local recurrence, with or without distant metastases, was confirmed by histological examination of biopsy specimens, additional surgery, or radiological studies.

### Preoperative bowel preparation and prophylaxis antibiotics

For elective laparoscopic left hemicolectomies, we used mechanical bowel preparation with Bowklean (magnesium oxide + sodium picosulfate + citric acid anhydrous) or polyethylene glycol (PEG) solutions on the day before surgery. Additionally, patients were asked to consume clear liquid diets on the day before surgery. Older patients and those with chronic kidney disease were prescribed with PEG solution.

Prophylactic antibiotics (cefazolin 1 g and metronidazole 500 mg) were prescribed within 60 min before the procedure. In prolonged surgery, additional intraoperative doses of cefazolin 1 g was given every 4 h once or twice.

### Operative techniques

The operator and their assistant performed all the procedures by standing on the right side of the patient. We applied a four port-laparoscopic approach. Pneumoperitoneum was created first, mostly via an umbilical opening with a needle or the mini-laparotomy method, depending on the surgeon’s preference and whether the patient had previously undergone abdominal surgery. After pneumoperitoneum, four ports were used with one 12-mm trocar at the umbilicus as a camera port, another 12-mm trocar at the right lower quadrant as a working port for the stapling device, and two additional 5-mm trocars in the right upper and left lower quadrants.

After all preparations and routine intra-abdominal examinations, we first performed a medial-to-lateral approach to mobilize the left colon and mesentery. Subsequently, we intended to perform a complete mesocolic excision and central vascular ligation. The left colic artery, left branch of middle colic artery, and inferior mesenteric vein were ligated using the Hem-o-lok system (Weck Closure Systems, Research Triangle Park, NC, USA) after completely dividing the mesentery of the planned resected bowel, including the marginal artery [11]. Colocolic anastomosis was performed using either EA or IA based on the surgeon’s preference.

In the EA group, the umbilical wound was extended to a length of 4–7 cm, based on the tumor size and colon mobilization, and a wound protector was used. An anastomosis was performed after pulling out the specimen via the umbilical wound. Colocolic anastomosis was created using either the antiperistaltic side-to-side stapler method or the isoperistaltic end-to-end hand-sewn method. In the IA group, we transected the colon laparoscopically with a linear staple, and anastomosis was performed using either the end-to-end hand-sewn method, isoperistaltic side-to-side double stapler method, or antiperistaltic side-to-side stapler method.

For the isoperistaltic staple method, enterotomies were performed on the antimesenteric side of both colons, followed by insertion of the jaws of the staples and anastomosis creation. The common enterotomy was closed laparoscopically by barbed suturing. For the antiperistaltic side-to-side stapler method, enterotomies were created near the transection side on both the proximal and distal colon, followed by side-to-side staple anastomosis, which was closed with a stapler. We then reinforced the crotch by additional suture. For both isoperistaltic and antiperistaltic side-to-side anastomosis methods, bleeding of the common channel will be examined before the common enterostomy was closed by barbed suture or staple.

After anastomosis, the specimen was extracted mainly through a Pfannenstiel incision in the IA group. For patients with a previous midline surgery, the umbilical wound was extended from the previous scar. Natural orifice specimen extraction (NOSE) was also an alternative choice for specimen extraction in the IA group. For the transrectal approach, we first irrigated the rectum with a povidone-iodine solution. An opening was made in the upper rectum, and a wound protector was inserted to protect and shorten the rectum. After specimen retrieval, the rectal opening was closed by suturing, and an air leak test was performed to confirm that there was no mechanically insufficient suture [12].

### Statistical analysis

All parameters were analyzed using the Statistical Package for Social Sciences (SPSS) version 24 (IBM Corp., Armonk, New York, USA). Categorical variables were compared using Pearson’s chi-squared test, whereas continuous variables were compared using the independent sample *t*-test and were expressed as means and standard deviations. Survival analysis was performed using the Kaplan-Meier curves and the log-rank test. Statistical significance was set at *p* < 0.05.

## Results

### Patient characteristics

**Fig. 1:**
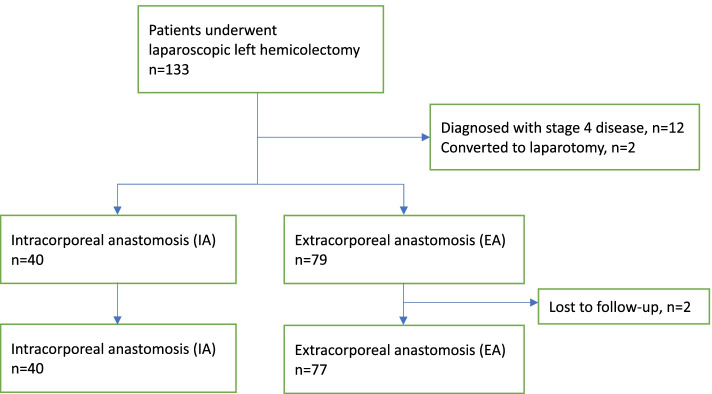
Flow chart of the patient selection process

Between July 2016 and September 2019, we retrospectively analyzed 133 consecutive patients who underwent laparoscopic left hemicolectomies. Twelve patients with distant metastasis were excluded. Of these 121 patients, two were converted to open surgery for extreme distension of the small intestine and colon (*n* = 1) and intestinal obstruction and severe adhesions (*n* = 1). Additionally, two EA patients were lost to follow-up. Of the remaining 117 patients, 40 and 77 underwent IA and EA, respectively. (Fig. 1) There were no significant differences in age, sex, BMI, preoperative albumin level, or comorbidities between two groups (Table 1).

**Table 1:** Patient characteristics and operative parameters of the intracorporeal and extracorporeal anastomosis groups

	IA^a^ (*n* = 40)	EA^b^ (*n* = 77)	*p*-value
Age (years)	61.45 ± 11.9	62.65 ± 13.5	0.637
Sex, *n* (%)			
Male	23 (57.5)	45 (58.4)	0.923
Female	17 (42.5)	32 (41.6)	
BMI^c^ (kg/m^2^)	23.92 ± 3.1	23.94 ± 4.6	0.977
Comorbidity, *n* (%)			
Hypertension	16 (40.0)	23 (29.9)	0.27
Cardiac disease	5 (12.5)	2 (2.6)	0.032
CVA^d^	0 (0)	4 (5.2)	0.142
Diabetes mellitus	10 (25)	9 (11.7)	0.064
Cirrhosis	1 (2.5)	1 (1.3)	0.634
ASA^e^ classification, *n* (%)			0.424
2	15 (37.5)	34 (44.2)	
3	25 (62.5)	41 (53.2)	
4	0 (0)	2 (2.6)	
Previous abdominal surgery, *n* (%)	6 (15.0)	18 (23.4)	0.287
Location, *n* (%)			
Transverse colon	10 (25)	29 (37.7)	0.387
Splenic flexure	5 (12.5)	8 (10.4)	
Descending colon	25 (62.5)	40 (51.9)	
Preoperative albumin	4.29 ± 0.3	4.19 ± 0.4	0.231
Operative time (mins)	240 ± 61.0	241 ± 68.9	0.906
Blood loss (mL)	33.7 ± 31.2	66.1 ± 201.9	0.397

*Abbreviations*: ^a^Intracorporeal anastomosis, ^b^Extracorporeal anastomosis, ^c^Body mass index, ^d^Cerebrovascular accident, ^e^American Society of Anesthesiologists

### Operative parameters

There were no significant differences in the operative time and blood loss between two groups (Table 1). For all left hemicolectomies with EA, the specimens were extracted through the midline by extending the original umbilical trocar wound. The IA approach had various options for specimen extraction, including extraction from the midline (17.5%, 7/40), the left lower abdominal wound (7.5%, 3/40), and the Pfannenstiel wound (35%, 14/40) and extraction with the NOSE method (40%, 16/40).

### Pathological parameters

There were no significant differences in tumor size, specimen length, nearest margin, number of harvested lymph nodes, stage of tumor invasion, or stage of lymph node invasion between the EA and IA groups. While no significant difference was observed in the harvested lymph nodes, more lymph nodes were harvested in the EA group than in the IA group (IA vs. EA: 25.0 vs. 32.2, *p* = 0.058). The EA group also had a more advanced pathological T stage than the IA group; however, this difference was not statistically significant (Table 2).

**Table 2:** Pathologic parameters of the intracorporeal and extracorporeal anastomosis groups

	IA^a^ (*n* = 40)	EA^b^ (*n* = 77)	*p*-value
p T stage			
T0	2 (5)	0 (0)	0.067
T1	9 (22.5)	12 (15.6)	
T2	6 (15)	10 (13)	
T3	22 (55)	40 (51.9)	
T4a	1 (2.5)	12 (15.6)	
T4b	0 (0)	3 (3.9)	
p N stage			
N0	30 (75)	50 (64.9)	0.25
N1	10 (25)	19 (24.7)	
N2	0 (0)	8 (10.4)	
TNM stage			0.179
Stage 0	2 (5)	0 (0)	
Stage 1	9 (22.5)	21 (27.3)	
Stage 2	18 (45)	29 (37.7)	
Stage 3	11 (27.5)	27 (35.1)	
Differentiation, *n* (%)			0.456
Well	6 (15)	6 (7.8)	
Medium	30 (75)	64 (83.1)	
Poor	4 (10)	7 (9.1)	
Angiolymphatic invasion, *n* (%)	9 (22.5)	18 (23.4)	0.915
Perineural invasion, *n* (%)	6 (15)	19 (24.7)	0.226
Tumor size (cm)			
Width	3.0 ± 1.8	3.5 ± 2.3	0.223
Length	2.7 ± 1.7	3.0 ± 1.6	0.409
Specimen length (cm)	17.7 ± 5.8	17.6 ± 5.9	0.951
Nearest margin (cm)	5.0 ± 2.3	4.8 ±1.9	0.593
Harvested lymph nodes (n)	25.0 ± 10.2	32.2 ± 22.3	0.058

*Abbreviations*: ^a^Intracorporeal anastomosis, ^b^Extracorporeal anastomosis

### Postoperative outcomes

Regarding short-term outcomes in the postoperative pain score category, no significant differences were observed in the NSR scale from the operation day to postoperative day 3 between the EA and IA groups. The amount of morphine used for pain control was not significantly different between two groups.

In the recovery category, better recovery was noted in the IA group as compared to the EA group. There were no significant difference in postoperative return of bowel function, such as first flatus passage (IA vs. EA: 2.1 vs. 2.5, *p* = 0.234) and first stool passage (IA vs. EA: 3.7 vs. 4.6, *p* = 0.067). However, there were significant differences in the time to tolerate liquid (IA vs. EA: 2.9 vs. 4.4, *p* = 0.006) and soft (IA vs. EA: 4.6 vs. 6.1, *p* = 0.007) diets. The mean length of postoperative stay was significantly shorter in the IA group than in the EA group (IA vs. EA: 5.7 vs. 7.1, *p* = 0.001) (Table 3).

**Table 3:** Short-term outcomes of the intracorporeal and extracorporeal anastomosis groups

	IA^a^ (*n* = 40)	EA^b^ (*n* = 77)	*p*-value
Pain scale, NRS^c^			
POD^d^ 0	4.5 ± 2.0	4.0 ± 1.8	0.158
POD^d^ 1	3.6 ± 1.8	4.0 ± 1.8	0.301
POD^d^ 2	2.8 ± 1.3	2.9 ± 1.5	0.665
POD^d^ 3	2.2 ± 1.0	2.3 ± 1.0	0.659
Morphine (mg)	10.6 ± 1.4	10.9 ± 1.6	0.906
Bowel movement (days)			
Time to first flatus passage	2.1 ± 1.1	2.5 ± 1.6	0.234
Time to first stool passage	3.7 ±2.3	4.6 ± 2.5	0.067
Tolerate liquid diet	2.9 ± 1.6	4.4 ± 3.0	0.006
Tolerate soft diet	4.6 ± 1.9	6.1 ± 3.1	0.007

Complications	1	7	0.565
Early morbidity, *n* (%)	0 (0)	6 (7.8)	0.07
Discharged morbidity, *n* (%)	1 (2.5)	1 (1.3)	0.634
Clavien-Dindo classification			
Grade 1	0	2	
Grade 2	1	3	
Grade 3	0	2	
Grade 4	0	0	
Grade 5	0	0	
Mortality	0	0	
Hospital stay (days)	5.7 ± 1.9	7.7 ± 3.4	0.001
POD^d^ 3 lab			
WBC^e^ (1000/uL)	10.3 ± 3.8	10.7 ± 3.4	0.853
Segment (%)	79.2 ± 8.3	76.4 ± 7.1	0.077
CRP^f^ (mg/L)	105 ± 70	70 ± 44	0.009

*Abbreviations*: ^a^Intracorporeal anastomosis, ^b^Extracorporeal anastomosis, ^c^Numeric rating scale, ^d^Postoperative day, ^e^White blood cell, ^f^C-reactive protein

Follow-up laboratory data on postoperative day 3 revealed that the IA group had more patients with higher CRP levels (IA vs. EA: 105 vs. 70, *p* = 0.009). There were no significant differences on postoperative day 3 white blood cell count (IA vs. EA: 10,316 vs. 10,719, *p* = 0.853) and segment (IA vs. EA: 79.2 vs. 76.4, *p* = 0.077) (Table 3).

Overall, no significant differences in complications were observed between the IA and EA groups during admission and after discharge. Among six out of 79 EA patients who experienced complications during admission, two had anastomosis leakage, with one requiring surgical intervention and the other receiving subsequent parenteral antibiotic treatment. One patient had postoperative bleeding and needed to be returned to the operating room for bleeding control. Other complications included chylus, ileus, and urine retention. After discharge, one EA patient exhibited ileus, and one IA patient had anastomosis bleeding. The IA patient with anastomosis bleeding was taking aspirin and clopidogrel for coronary artery disease, and the bleeding episode occurred 10 days after discharge (Table 3).

**Fig. 2:**
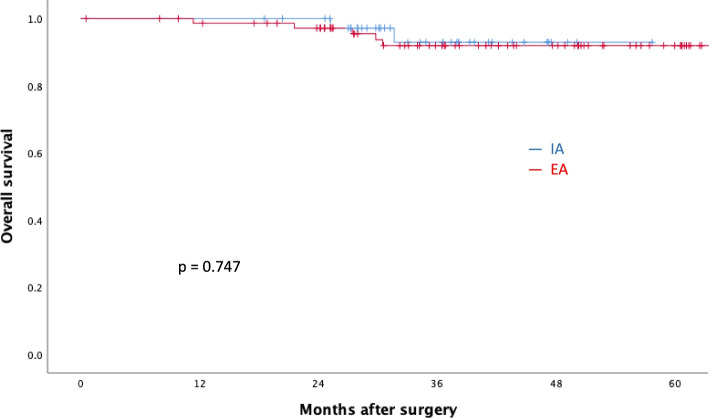
Two-year overall survival rates of the IA and EA groups. Blue bars, intracorporeal anastomosis (IA); red bars, extracorporeal anastomosis (EA)

**Fig. 3:**
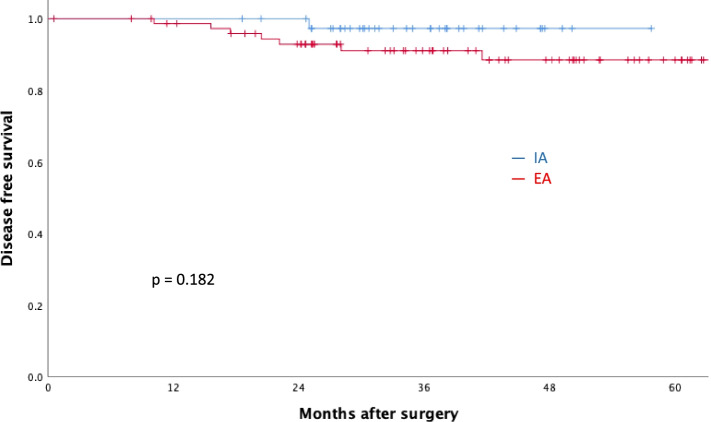
Two-year disease-free survival rates of the IA and EA groups. Blue bars, intracorporeal anastomosis (IA); red bars, extracorporeal anastomosis (EA)

The median follow-up periods were 35.4 and 40.2 months in the IA and EA groups, respectively. The 2-year overall (IA vs. EA: 95.0% vs. 93.5%, *p* = 0.747, Fig. 2) and disease-free (IA vs. EA: 97.5% vs. 90.9%, *p* = 0.182, Fig. 3) survival rates were not significantly different between two groups.

In the subgroup analysis, one peritoneal carcinomatosis recurred in the IA group on the 25th month after surgery (Table 4). There were 7 recurrences in the EA group, 3 of which were peritoneal carcinomatosis. The disease-free survival rate regarding peritoneal recurrence was not different between two groups at the end of the 2-year follow-up period.

**Table 4:** Medium-term outcomes of the intracorporeal and extracorporeal anastomosis groups

	IA^a^ (*n* = 40)	EA^b^ (*n* = 77)	*p*-value
Medium follow-up (months)	35.4 ± 8.9	40.2 ± 16.1	0.068
Recurrence, *n* (%)	1 (2.5)	7 (9.1)	0.18
Liver	0 (0)	5 (6.5)	0.1
Lung	0 (2)	2 (2.6)	0.304
Carcinomatosis	1 (2.5)	3 (3.9)	0.693
Death, *n* (%)	2 (5)	5 (6.5)	0.747
2-year disease-free survival (%)	97.5	90.9	0.182
Survival (%)	38 (95)	72 (93.5)	0.747

*Abbreviations*: ^a^Intracorporeal anastomosis, ^b^Extracorporeal anastomosis

## Discussion

This study compared the short- and medium-term outcomes between EA and IA in laparoscopic left hemicolectomy for colon cancer. The study represents the first report on the specimen quality of laparoscopic left hemicolectomy with IA and EA. There were no differences in early complications (*p* = 0.07), late complications (*p* = 0.634), or mortality between the EA and IA groups. Additionally, there was no difference in the medium-term outcomes between two groups. Our results suggested that IA was safe and feasible for laparoscopic left hemicolectomy. This approach had a faster resumption of liquid and soft diets and shorter hospital stays than those of EA and acceptable short-term morbidity and medium-term outcomes.

Several studies have established the feasibility and safety of laparoscopic colonic resection using the open method [13, 14], while some studies have shown the advantages of the laparoscopic approach [15–18]. Most studies that compared IA and EA methods mainly focused on the right side of the colon. Only a few studies have compared these two anastomosis techniques for colon cancer located in the distal transverse, splenic flexure, and descending parts of the colon. Theoretically, laparoscopic left colectomy is more challenging than laparoscopic right colectomy. Anastomosis in left colectomy requires extended mobilization of the two fixed colon ends to prevent tension, as the terminal ileum is much easier to mobilize in laparoscopic right colectomy.

In this study, the IA group demonstrated a shorter hospital stay, which might be partially contributed by the IA group’s substantially earlier resumption of liquid and soft diets, despite comparable postoperative pain and complications between the EA and IA groups. Swaid et al. also reported a shorter hospital stay for the IA group. Furthermore, the IA group included younger participants, and surgeons tended to discharge young patients earlier than older patients [19].

Although the median times to first flatus (IA vs. EA: 2.1 days vs. 2.5 days) or first stool passage (IA vs. EA: 3.7 days vs. 4.6 days) appeared shorter in the IA group than in the EA group, these differences were not statistically significant. The IA group showed faster resumption of liquid and soft diets than the EA group in this study, which is consistent with findings from other reports [19–21] and our previous study on right hemicolectomy with IA [22]. This finding might result from less tissue manipulation and minor bowel mobilization during colectomy and anastomosis in the IA group.

There was no significant difference in the postoperative pain scores between the IA and EA groups in our study. In the EA group, the bowel stump was pulled out of the small midline minilaparotomy wound onto the body surface of the abdominal wall. Creating a better surgical field for anastomosis might require more extensive incisional wounds to achieve secure anastomosis. In contrast, the incision wounds in the IA group were only created for specimen extraction and did not need to be used for anastomosis. As such, the mini-laparotomy wound in the IA group was shorter than in the EA group.

Since the IA method has several options to select the location of the mini-laparotomy incision, any abdominal site can be used to remove the specimen. Our study showed that, in addition to the midline incision, the left lower quadrant, Pfannenstiel incision, and the NOSE method were utilized for specimen extraction in the IA group. The advantages of Pfannenstiel wounds include less pain, better cosmetic results, and a lower incidence of incisional hernias than midline wounds [23, 24]. However, during our 2-year follow-up, no incisional hernia was found in either group. Additionally, 16 patients in the IA group had specimens extracted using the NOSE method, which might have resulted in less postoperative pain. Nonetheless, postoperative pain did not differ significantly between the two groups in this study.

The shorter hospital stays in the IA group might be attributed to fewer postoperative complications in the IA group than in the EA group (IA vs EA: *n* = 0 vs. *n* = 6, *p* = 0.07). However, this difference was not statistically significant. The leakage rate should be carefully considered when comparing these two anastomosis techniques. In this regard, no difference in the leakage rates was observed between two groups in this study. However, two EA patients suffered from anastomotic leakage, with one requiring surgical intervention. In this case, the second surgery with laparoscopic exploration on postoperative day 11 showed a 0.5-cm gap over the angle between the two colon stumps. The other patient with anastomotic leakage received only conservative treatment, including non-oral feeding and total parenteral nutrition support, and was discharged approximately 1 month postoperatively. While several studies have compared leakage rates between these two types of anastomosis in right colectomy, no significant difference was observed [9, 10, 25]. Few analyses of left colectomy also showed no significant differences in leakage rates [19, 21].

Several studies have compared the IA and EA methods in laparoscopic left colectomy. However, none has reported postoperative laboratory data, with the exception of Masubuchi et al., who noted that the serum C-reactive protein level was higher in the IA group [21]. In this study, we observed elevated serum C-reactive protein levels in the IA group on postoperative day 3, which might result from bowel opening in the IA group during surgery. Despite high levels of inflammatory markers in the IA group, postoperative complications, such as intra-abdominal abscess formation or wound infection, were not higher in the IA group than in the EA group. Based on our experience, routine laparoscopic lavage with water after IA creation is an effective and safe practice.

There were no differences in surgical parameters, including operation time and blood loss, between the IA and EA groups. The IA method is rather challenging, particularly during the learning curve, which may require more hands-on time [26]. In other studies, operative time tended to be longer in the IA group [27, 28]. In 2016, we started to perform left colectomy using the IA method in our hospital. However, this approach was first utilized for right hemicolectomy before being adopted for left colectomy. Therefore, our accumulated experience in right hemicolectomy with IA might result in a shorter learning curve for IA in laparoscopic left hemicolectomies.

Although the number of lymph nodes harvested on pathological examination was higher in the EA group than in the IA group, the difference was not statistically significant in our study (IA vs. EA: 25 vs. 32.2, *p* = 0.058). Similar to other studies, the mean numbers of harvested lymph nodes in both groups were higher than the recommended number (12). Notably, no previous study has compared the specimen length and the nearest margin to the tumor. Grieco et al. observed no significant difference in specimen length (25.7 cm vs. 29.0 cm, IA vs. EA, *p* = 0.39) between the two groups in left colectomy for splenic flexure cancer [20]. While Grieco et al. showed longer specimens than ours, both IA and EA groups in our study had more harvested lymph nodes than the recommended number, which is an important sign of specimen quality (the numbers of harvested lymph nodes in Grieco et al. were 19 ± 10.1 vs. 17.0 ± 5.5, IA vs. EA). Our study demonstrated no significant differences between the EA and IA groups in specimen length and the nearest tumor margin.

Previous studies have compared the short-term outcomes of laparoscopic left colectomy using IA and EA methods. However, no studies have examined long-term survival, such as disease-free survival. Approximately, 95% of colorectal cancer recurs within 5 years after radical surgery, and in the majority of the cases, tumor recurs within 2 years [29, 30]. In this study, we followed these patients for at least 2 years after surgery, with a median follow-up time of 38.5 months. Our preliminary medium-term outcome results showed that the disease-free survival rates of both groups were comparable.

During follow-up, seven EA patients relapsed as compared to one in the IA group. A major concern of the IA approach was the possibility of leakage of tumor cells via bowel opening during anastomosis. There was one case (1/40) of peritoneal carcinomatosis in the IA group and three (3/77) in the EA group, with no statistically significant difference. This result indicated that the risk of tumor cell dissemination during anastomosis in the abdominal cavity was not increased. However, a longer follow-up period is still required to determine whether the long-term outcomes of these two anastomosis methods are comparable.

To our best knowledge, along with the comparison of specimen lengths and resection margins, our study is the first to report the interim results of IA and EA methods. While we intraoperatively opened the bowel during the left hemicolectomy using the IA approach, peritoneal recurrence did not increase with IA surgery at the end of the 2-year follow-up.

Our study had some limitations regarding its retrospective design, the small sample size for each study group, and the requirement for a longer follow-up duration to improve the reliability of our findings. Another limitation of this study was possible selection bias for the anastomosis type and postoperative care. In this regard, there were no specific criteria for IA or EA selection in left hemicolectomies, and the selection of anastomosis method was at the attending surgeon’s discretion. Additionally, while there were no differences in postoperative care strategies between the IA and EA groups, we did not have standard guidelines for postoperative care, including those related to postoperative diets, which was based on the attending physician’s preference.

## Conclusions

This study demonstrated that laparoscopic left colectomy with IA was technically feasible with better short-term outcomes, including shorter hospital stays and faster resumption of liquid or soft diets. Although the IA group had a higher postoperative serum C-reactive protein level, no complications were observed. The medium-term outcomes regarding overall survival and disease-free survival were comparable between the IA and EA procedures. However, additional case studies are required to confirm this finding, and a longer follow-up period is needed to determine whether the long-term survival is similar between these two anastomosis techniques.

## Data Availability

The datasets generated and analyzed during the current study are available from the corresponding author on reasonable request.

## Abbreviations

BMI: Body mass index
CEA: Carcinoembryonic antigen
CRP: C-reactive protein
EA: Extracorporeal anastomosis
IA: Intracorporeal anastomosis
NOSE: Natural orifice specimen extraction
NRS: Numeric rating scale
PEG: Polyethylene glycol
